# Impact of Combined Treatment with ARNi and SGLT2i on Clinical and Echocardiographic Outcomes in Patients with CRT During Mid-term Period

**DOI:** 10.2174/011573403X350660250203111206

**Published:** 2025-03-14

**Authors:** Tariel A. Atabekov, Mikhail S. Khlynin, Sergey N. Krivolapov, Roman E. Batalov, Sergey V. Popov

**Affiliations:** 1 Cardiology Research Institute, Tomsk National Research Medical Center, Russian Academy of Sciences, Kievskaya st., 111a, Tomsk, 634012, Russian Federation

**Keywords:** Heart failure, cardiac resynchronization therapy, angiotensin receptor neprilysin inhibitors, sodium-glucose co-transporter 2 inhibitors, clinical outcomes, echocardiographic outcomes

## Abstract

**Introduction:**

Sodium-glucose co-transporter 2 inhibitors (SGLT2i) and angiotensin receptor neprilysin inhibitors (ARNi) are new classes of medications with an evolving role in heart failure (HF) patients. However, the effect of combining these drugs with cardiac resynchronization therapy (CRT) remains less certain.

**Objective:**

This study aimed to investigate the impact of combined treatment with ARNi and SGLT2i on clinical and echocardiographic outcomes in CRT patients during 12-month follow-up.

**Methods:**

HF patients with CRT implantation indications were enrolled in the non-randomized and retrospective study and were grouped in no ARNi and SGLT2i (1^st^ group) and combined treatment with ARNi and SGLT2i (2^nd^ group) cohorts. The CRT response criteria were as follows: improvement of NYHA class ≥1 and left ventricular end-systolic volume reduction ≥15% or left ventricular ejection fraction improvement ≥5% from the baseline during the 12-month follow-up.

**Results:**

A total of 52 patients were included. At the 12-month follow-up, 18 of 35 (51.4%) patients in the 1^st^ group and 16 of 17 patients (94.1%) in the 2^nd^ cohort met CRT responder criteria (*p=*0.002). In multivariable logistic regression, combined treatment with ARNi and SGLT2i [odds ratio (OR): 20.09; 95% confidence interval (CI): 2.10-192.15; *p=*0.009] and non-ischemic HF (OR 5.51; 95% CI 1.21-24.91; *p=*0.026) were associated with CRT response.

**Conclusion:**

The combined treatment with SGLT2i and ARNi in patients with CRT improved the echocardiographic and clinical outcomes during the 12-month follow-up. In our study cohort, the CRT response was associated with non-ischemic HF and combined treatment with ARNi and SGLT2i.

## 1 INTRODUCTION

### 1.1 Background

The heart failure (HF) remains a serious public health problem and affects 64 million people worldwide [1]. Although advances in medical therapy and device assistance have significantly improved HF outcomes, the implications of HF are still significant [2].

In recent years, innovative developments have been made in HF treatment and management, according to robust evidence from several trials of sodium-glucose co-transporter 2 inhibitors (SGLT2i) and angiotensin receptor neprilysin inhibitors (ARNi) [3-6]. The studies have shown the treatment alone with ARNi or SGLT2i or their combination to improve the left ventricular end-systolic volume (LVESV) and ejection fraction (LVEF). According to current guidelines, the treatment with ARNi alone or in combination with SGLT2i outperforms mineralocorticoid receptor antagonists and beta-adrenergic blockers [7].

Moreover, the therapy with the most proven effectiveness in HF patients with a reduced LVEF is сardiac resynchronization therapy (CRT) [8]. However, the lack of positive response to CRT as an absence of reverse left ventricle remodeling, resulting in no improvement in symptoms, hospitalization for HF, or mortality, still ranges up to 30-50% [9].

### 1.2 Rationale and Knowledge Gap

To date, many attempts have been made to find the reasons for non-response to CRT. The clinical advantage of SGLT2i and ARNi monotherapy or combined treatment is evident in non-CRT device wearers [3, 4, 6, 10-12]. Currently, there are insufficient data evaluating the impact of the combined treatment with SGLT2i and ARNi in CRT recipients [13, 14]. Thus, there is limited evidence of the additional benefit of combined SGLT2i and ARNi treatment in CRT patients, even if initiated after device implantation.

### 1.3 Objective

The aim of the study was to investigate the impact of combined treatment with ARNi and SGLT2i on clinical and echocardiographic outcomes in patients with CRT during mid-term follow-up.

## 2 MATERIALS AND METHODS

### 2.1 Study Design and Participants

**Fig. (1):**
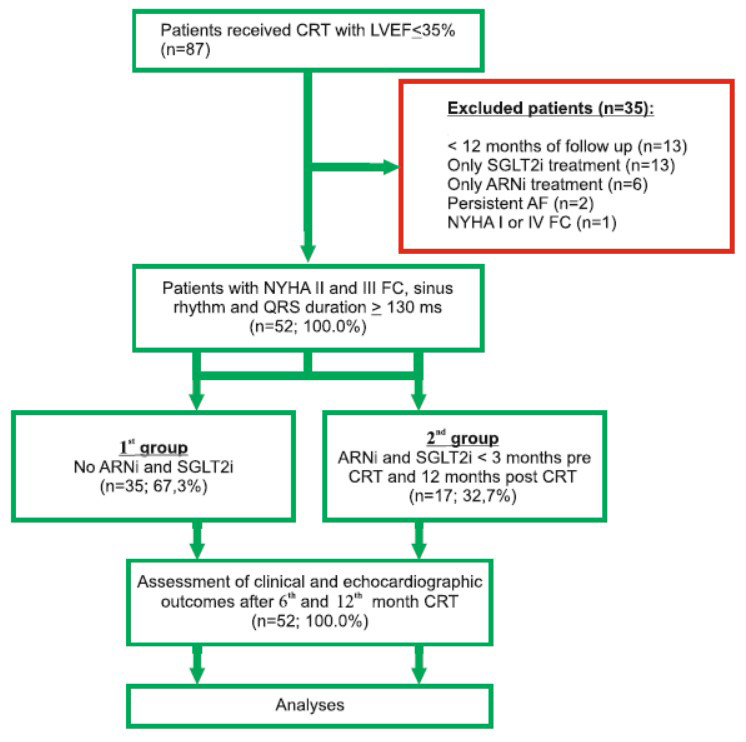
Schematic description of study enrollment flow and design. **Abbreviations:** AF: atrial fibrillation, ARNi: angiotensin receptor neprilysin inhibitor, CRT: cardiac resynchronization therapy, FC: functional class, LVEF: left ventricle ejection fraction, NYHA: New York Heart Association, SGLT2i: sodium glucose co-transporter 2 inhibitor.

This was a single-center retrospective clinical non-randomized observational study that included patients with LVEF ≤ 35% who underwent CRT devices with the defibrillator (CRT-D) implantation in the Department of Surgical Arrhythmology and Cardiac Pacing, Cardiology Research Institute, from November 2017 to December 2021. Patients with age below 18, New York Heart Association (NYHA) I and IV functional class (FC) of HF, hypertrophic cardiomyopathy, persistent atrial fibrillation (AF), lack of full clinical information at 12-months follow-up, and individuals with only SGLT2i or ARNi treatment prior to CRT-D implantation were excluded (Fig. **1**). Therefore, we analyzed remaining patients with NYHA II and III FC of HF and QRS duration ≥ 130 ms, who were divided into no ARNi and SGLT2i treatment (1^st^ group) and combined treatment with ARNi and SGLT2i (2^nd^ group) cohorts. We classified individuals who did not meet the combined response to CRT criteria after 12 months as non-responders and individuals who met the criteria as responders. The SGLT2i used in our study was dapagliflozin 10 mg and ARNi was Uperio (valsartan + sacubitril) with an average dose of 111.7 ± 33.2 mg.

We considered the criteria for ischemic HF to be the presence of myocardial infarction in anamnesis or prior revascularization and/or ≥50% stenosis in ≥1 of the coronary arteries according to coronary artery angiography.

The following data were collected: patient’s history, demographic indicators, risk factors for cardiovascular disease, pharmacotherapy data, NYHA FC, results of 6-minute walk distance test (6MWDT), underlying heart disease, electrocardiographic and echocardiographic measurements, CRT-D implantation and device interrogation data.

### 2.2 Definition of CRT Response Criteria

The CRT response criteria were as follows: absence of hospitalization related to HF, improvement of NYHA class ≥1 (clinical response), and LVESV reduction ≥15% or LVEF improvement ≥5% (echocardiographic response) from the baseline during 12-month follow-up [15, 16]. The presence of clinical and echocardiographic response was defined as a combined response to CRT.

### 2.3 Endpoints

The primary study endpoint was the evaluation of the combined CRT response at 12 months follow-up. The secondary study endpoints were cardiovascular HF-related deaths, HF hospitalization, CRT-D registered arrhythmic events, and appropriate ICD therapy events.

### 2.4 Consent

The standards of the Helsinki Declaration and Good Clinical Practice were followed in our study. The study was approved by the Cardiology Research Institute’s local ethics committee (protocol no. 163, dated November 08, 2017). The majority of enrolled CRT recipients are registered in ClinicalTrials.gov (NCT03667989). The consent for the publication of clinical data was obtained from all the subjects.

### 2.5 Acquisition and Analysis of 6MWDT Results

The FC of HF was assessed using 6MWDT in conformity with the NYHA criteria:

- ≥551 m – patient without HF signs;

- 426-550 m – I FC of HF;

- 301-425 m – II FC of HF;

- 151-300 m – III FC of HF;

- ≤150 m – IV FC of HF.

### 2.6 Electrocardiogram Acquisition and Analysis

Prior to and post CRT-D implantation, the standard 12-lead electrocardiograms (EGM) (25 mm/s speed; 10 mm/mV amplitude) from the supine position were recorded. The definition of complete LBBB included the Strauss criteria [17]. The following information was collected: bundle branch block morphology, alpha angle, intervals [PQ, corrected QT (QT_c_) adjusted for LBBB or biventricular pacing (BP)] [18], baseline and 12-month QRS duration, and a notch in QRS in leads I, aVL, V_5_, V_6_, QS or rS in V_1_ and V_2_ [17].

### 2.7 Echocardiographic Acquisition and Analysis

Transthoracic echocardiography (TTE) was assessed using an ultrasound machine (Philips HD15 PureWave, Netherlands) prior to and post-12 months CRT-D implantation. The TTE was carried out from standard echocardiographic positions with the determination of the LVEF and heart chamber volumes and dimensions. The aortic, mitral, and tricuspid valve functions, and the right ventricle and LV contractility were evaluated. TTE was carried out according to current recommendations [19].

### 2.8 CRT Device Implantation and Programming

The atrial and defibrillation leads were implanted at the right atrial appendage and right ventricular septum or apex, respectively. The LV leads were placed in one of the target veins (lateral, posterolateral, or anterolateral). Lead implantation was performed by employing a transvenous approach under fluoroscopic guidance according to a conventional implantation procedure. Lead positions were confirmed in postero-anterior and left anterior oblique fluoroscopy view and intra-operative threshold testing. The lead parameters (sensing amplitude, capture test, and impedance) were assessed.

Programming of the CRT-D was performed in accordance with the current guidelines and standards [20]. The “monitoring” or “monitor only” zone was set with a heart rate of 150–180 beats per minute (bpm) (>50 consecutive cycles) without device therapy [anti-tachycardia pacing (ATP) and shock]. The ventricular tachycardia (VT) and fibrillation (VF) zones were programmed for 180-240 bpm with 30 cycles and for ≥ 240 bpm with 12 cycles, respectively, in accordance with standard device therapy for each zone.

### 2.9 Statistical and Survival Analysis, Risk Stratification, and Score Development

Categorical and qualitative variables were presented as numbers (n) and percentages (%), normally distributed continuous variables as mean (M) ± standard deviation (SD), and non-normally distributed variables as median (M_e_) and interquartile ranges (Q_1_; Q_3_). The distribution of continuous data was tested for normality using Kolmogorov-Smirnov, Lilliefors, and Shapiro-Wilk tests. Group differences in continuous data were analyzed using the two-sided Student's t-test for normally distributed data or the Mann–Whitney U-test for independent ordinal or non-normally distributed data. For dependent samples, the Wilcoxon test was used. The distribution of categorical and qualitative variables was analyzed using Fisher's exact or chi-square test.

For the primary outcome, logistic regression with stepwise elimination was used to distinguish the possible predictors of combined CRT response. We first performed a univariable logistic regression analysis to test the association between our primary endpoint (dependent variable) and all clinical outcomes, including the combined treatment with SGLT2i and ARNi (independent variables). Characteristics significantly (*p* < 0.05) related to the outcome according to univariable logistic regression were first introduced as potential variables in a multivariable logistic regression analysis. The test for collinearity was performed to exclude possible confounders between included independent variables. Goodness-of-fit was assessed using the Hosmer-Lemeshow test. Correlations with significance among the predictors and other parameters were assessed using a t-test and Pearson’s test. Our regression analysis results were presented as odds ratios (OR) with 95% confidence intervals (CI).

Finally, variables independently related to our endpoint were entered into the risk score analysis. The area under the curve (AUC) was calculated to estimate the distinguishing ability of the risk stratification model. In addition, to examine the risk of the primary and secondary outcomes, we used Kaplan-Meier curves to assess the outcome-free survival function stratified by the hypothesis of combined treatment with ARNi and SGLT2i.

All statistical analyses were carried out using the statistical software Medcalc 19.2.6 (USA) and Statistica 10.0, StatSoft (USA), and statistical significance was determined by a *p*-value < 0.05.

## 3 RESULTS

### 3.1 Study Population

Of 87 patients with HF and indication for CRT, we excluded 35 patients with an incomplete 12-month follow-up (n=13), only SGLT2i medication (n=13), only ARNi medication (n=6), persistent AF (n=2), or NYHA I and IV FC (n=1). Of the remaining 52 (100.0%) CRT recipients, the average age was 60.0 ± 10.0 years, and 34 (65.4%) individuals were males. The first group included 35 (67.3%) patients without ARNi and SGLT2i treatment. The second group included 17 (32.7%) patients with combined ARNi and SGLT2i treatment.

The patients’ demographic and clinical characteristics are presented in Table **1**. Individuals in the no ARNi and SGLT2i treatment group did not differ significantly from those in the combined ARNi and SGLT2i treatment group with respect to age, gender, body mass index, HF etiology, or comorbities. Likewise, no significant differences in pre-CRT arrhythmias, and dyspnea, assessed with NYHA classification, baseline ECG and TTE, LV lead position, and BP rate after 12 months of CRT could be observed. The cohorts did not differ significantly regarding medication on admission. With the exclusion of the angiotensin-converting enzyme inhibitors (ACEi) and angiotensin II receptor blockers (ARB), formally statistically significant differences were observed (*p<*0.001 and *p=*0.002, respectively). These significant distinctions were due to the fact that patients with combined ARNi and SGLT2i treatment did not take ACEi or ARB.

### 3.2 Electrocardiographic Characteristics

Pre and post-CRT, ECG indicators (PQ, QRS, QT_c_, alpha angle, or bundle branch block morphology) did not differ significantly in both groups with the exception of QT_c_ interval adjusted for LBBB/BP. For the baseline QT_c_, there was a formally statistically significant difference (*p=*0.019). Likewise, significant differences in 6-month CRT could be observed (442.0 ± 28.9 ms *vs.* 419.0 ± 28.9 ms, *p=*0.004). At the 12-month follow-up, the groups did not differ in this indicator (442.2 ± 28.9 ms *vs.* 431.4 ± 35.3 ms, *p=*0.359).

**Fig. (2):**
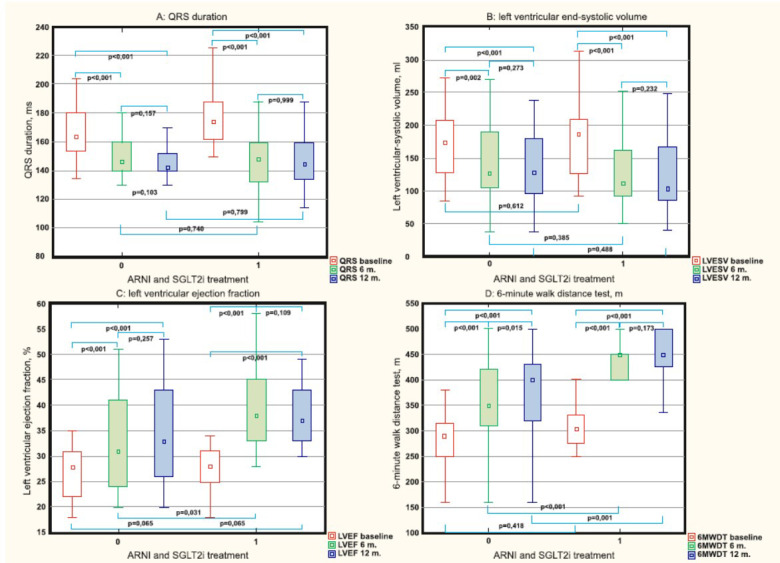
Effectiveness of sodium-glucose co-transporter 2 inhibitors (SGLT2i) and angiotensin receptor neprilysin inhibitor (ARNi) in (**A**) QRS duration, (**B**) left ventricular end-systolic volume (LVESV), (**C**) left ventricular ejection fraction (LVEF), and (**D**) 6-minute walk distance test (6MWDT) changes at baseline and 6 and 12 months follow-up. 0 means no ARNi and SGLT2i group, 1 indicates the combined ARNi and SGLT2i treatment group.

At 6^th^ and 12^th^ months follow-up, QRS duration in both groups decreased significantly [no ARNi and SGLT2i group: 164.0 ms (154.0; 180.0) *vs.* 146.0 ms (140.0; 160.0) and 142.0 ms (140.0; 152.0) (*p<*0.001); ARNi and SGLT2i treatment group: 174.0 ms (162.0; 188.0) *vs.* 146.0 ms (140.0; 160.0) and 142.0 ms (140.0; 152.0) (*p<*0.001)] (Fig. **2A**). A comparison of QRS duration at baseline and 6^th^ and 12^th^ months between the groups did not reveal significant differences.

### 3.3 Echocardiographic Characteristics

At the 6^th^ and 12^th^ months follow-up, both groups showed significant improvement in the basic echocardiographic indicators. Thus, in no ARNi and SGLT2i group, LVESV significantly decreased from 175.0 ml (128.0; 208.0) to 127.0 ml (105.0; 190.0) (*p=*0.002) and 129.0 ml (96.0; 180.0) (*p<*0.001); in ARNi and SGLT2i group, it decreased from 186.0 ml (126.0; 210.0) to 112.0 ml (93.0; 163.0) (*p<*0.001) and 104.0 ml (86.0; 167.0) (*p<*0.001), respectively (Fig. **2B**). However, a comparison of 6^th^ and 12^th^-months ∆LVESV between groups [8.8% (0.0; 37.1) *vs.* 26.2% (12.5; 42.6) and 17.3% (0.0; 37.1) *vs.* 28.3% (14.1; 44.0), respectively] did not reveal significant differences (*p=*0.062 and *p=*0.101, respectively).

In no ARNi and SGLT2i group, at the 6^th^ and 12^th^ month follow-up, LVEF significantly increased from 28.0% (22.0; 31.0) to 31.0% (24.0; 41.0) (*p<*0.001) and 32.0% (26.0; 43.0) (*p<*0.001); in ARNi and SGLT2i group, it increased from 28.0% (25.0; 31.0) to 38.0% (33.0; 45.0) (*p<*0.001) and 37.0% (33.0; 43.0) (*p<*0.001) (Fig. **2C**). A comparison of 6^th^-month ∆LVEF between groups [14.2% (0.0; 38.7) *vs.* 31.8% (20.5; 58.0), respectively] did not reveal significant differences (*p=*0.059). However, ∆LVEF at the 12^th^ month in no treatment group was significantly lower than in the ARNi and SGLT2i combined treatment group [16.6% (3.2; 45.7) *vs.* 32.0% (22.5; 66.6), *p=*0.0^45^].

### 3.4 Clinical Findings at 6^th^ and 12^th^ Month of Follow-up After CRT

Patients from both groups showed an improvement in the 6MWDT indicator at the 6^th^ and 12^th^ month follow-up. In no treatment group, 6MWDT improved from 290.0 m (250.0; 310.0) to 350.0 m (310.0; 420.0) and 400.0 m (320.0; 430.0) (*p<*0.001), and in ARNi and SGLT2i treatment group, it improved from 320.0 m (275.0; 350.0) to 450.0 m (400.0; 450.0) and 450.0 m (426.0; 500.0) (*p<*0.001), respectively (Fig. **2D**). It should be taken into account that in no treatment group, the 6MWDT was significantly lower than in combined SGLT2i and ARNi treatment cohort at the 6 (*p<*0.001) and 12 (*p=*0.001) months follow-up.

**Fig. (3):**
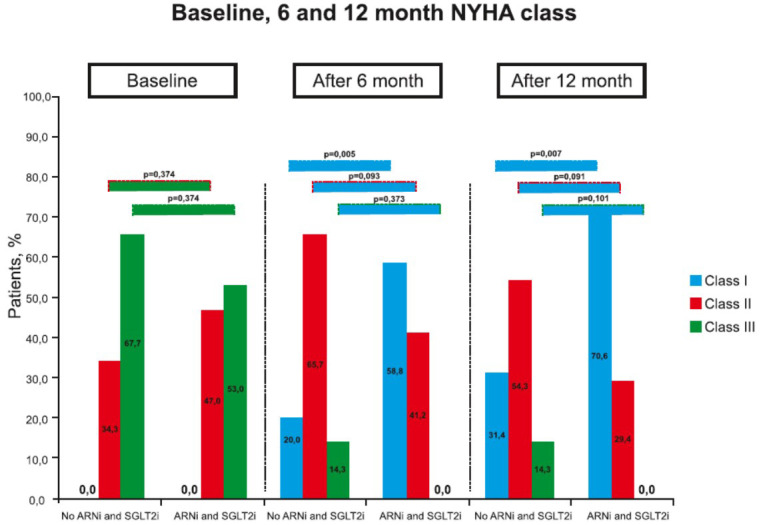
Effectiveness of sodium-glucose co-transporter 2 inhibitors (SGLT2i) and angiotensin receptor neprilysin inhibitor (ARNi) in New York Heart Association (NYHA) functional class variation at baseline and 6^th^ and 12^th^-months follow-up in the overall population.

After 6^th^ months of CRT, in the no treatment group, NYHA class improvement was achieved in 23 (65.7%) patients, while in the combined ARNi and SGLT2i treatment group, NYHA class improvement was revealed in 17 (100.0%) (*p=*0.005). At the 12^th^ month of follow-up, improvement in NYHA class in the 1^st^ group was observed in 26 (74.3%) patients, and in the 2^nd^ group, it was observed in 17 patients (100.0%) (*p=*0.021). It should be noted that there were significantly fewer patients with NYHA class I in the no treatment group at 6^th^ (*p=*0.005) and 12^th^ (*p=*0.007) months follow-up compared to the group with combined ARNi and SGLT2i treatment (Fig. **3**).

**Fig. (4):**
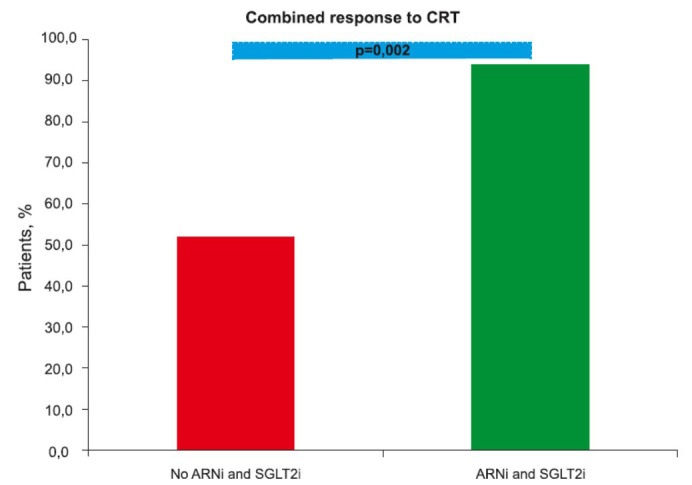
Combined (clinical and echocardiographic) response to cardiac resynchronization therapy (CRT) at 12^th^ months of follow-up in no angiotensin receptor neprilysin inhibitor (ARNi) and sodium-glucose co-transporter 2 inhibitor (SGLT2i) and ARNi and SGLT2i combined treatment groups.

According to the combined CRT response definition at the 12^th-^month follow-up, 18 of 35 (51.4%) individuals in the 1^st^ cohort and 16 of 17 (94.1%) in the 2^nd^ one were CRT responders (*p=*0.002) (Fig. **4**).

### 3.5 Risk Stratification Analysis

**Fig. (5):**
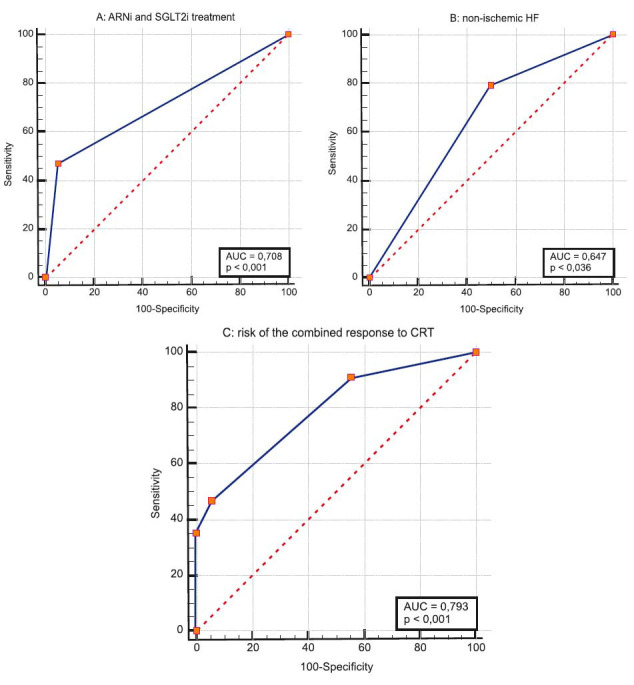
ROC curves to assess (**A**) the ability of combined sodium-glucose co-transporter 2 inhibitors (SGLT2i) and angiotensin receptor neprilysin inhibitor (ARNi) treatment, (**B**) non-ischemic heart failure (HF), and (**C**) the entire risk model to discriminate CRT recipients with and without combined response.

According to the estimated parameters, combined ARNi and SGLT2i treatment, non-ischemic HF, and baseline QTc interval adjusted for LBBB were the variables most closely associated with the combined response to CRT (Fig. **5**). Their distinguishing ability was evaluated by ROC analysis showing an AUC of 0.708 (95% CI: 0.565 to 0.825), 0.647 (95% CI: 0.502 to 0.775), and 0.640 (95% CI: 0.495-0.768), respectively (Figs. **5A** and **5B**).

**Fig. (6):**
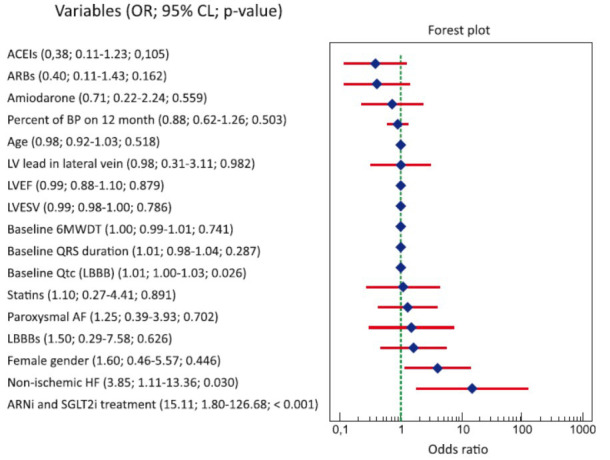
Forest plot showing the univariable logistic regression results. **Abbreviations:** 6MWDT: 6-minute walk distance test, ACEIs: angiotensin-converting enzyme inhibitors, AF: atrial fibrillation, ARBs: angiotensin II receptor blockers, ARNi: angiotensin receptor neprilysin inhibitor, BP: biventricular pacing, 95% CI: 95% confidence interval, HF: heart failure, LBBBs: left bundle branch block meeting Strauss criteria, LV: left ventricle, LVEF: left ventricular ejection fraction, LVESV: left ventricular end-systolic volume, OR: odds ratio, QT_c_ (LBBB): corrected QT_c_ interval adjusted for LBBB, SGLT2i: sodium-glucose co-transporter 2 inhibitor.

The univariable logistic regression showed combined ARNi and SGLT2i treatment (OR=15.11; 95% CI 1.80-126.68; *p<*0.001), non-ischemic HF (OR=3.85; 95% CI 1.11-13.36; *p=*0.03), and baseline QT_c_ interval adjusted for LBBB (OR=1.01; 95% CI 1.00-1.03; *p=*0.026) to be independently associated with the combined response to CRT (Fig. **6**).

Only ARNi and SGLT2i treatment variable (OR=20.09, 95% CI: 2.10-192.15, *p=*0.009) and non-ischemic HF parameter (OR=5.51, 95% CI: 1.21-24.91, *p=*0.026) remained significant in multivariable regression, even after adjusting for gender, age, baseline QRS duration, LVEF, LVESV, LV lead lateral position, percent of BP on 12^th^ month CRT and paroxysmal AF. The beta coefficients in the logistic equation corresponded to the natural logarithm of the OR [ln (OR)=β; OR=e^β^].

### 3.6 Score Development

Based on the collected data, a risk score for the combined response to CRT was set using logistic regression analysis. The combined treatment with ARNi and SGLT2i and non-ischemic HF variables were inserted in the final risk model since these indicators remained significant in multivariable logistic regression analysis, even after adjusting for gender, age, baseline QRS duration, LVEF, LVESV, LV lead lateral position, percentage of BP at 12 month CRT, and paroxysmal AF. The AUC was calculated to assess the distinguishing ability of the risk stratification model. The risk score showed distinguishing ability, which was evidenced by an AUC of 0.793 (Fig. **5C**). At a cutoff value of > 0.63, the risk model demonstrated a sensitivity of 47.1% and a specificity of 94.4% in discernment in patients with LVEF ≤ 35% who underwent CRT-D implantation regarding the occurrence of the combined response to CRT. Our risk stratification analysis showed a positive predictive value of 91.2% and a negative predictive value of 44.4%.

The combined treatment with ARNi and SGLT2i and non-ischemic HF were indicated in the score equation by assigning a value of 1 (apparent) or 0 (in-apparent), respectively. The result of the logistic equation below is the probability (p) of the combined response to CRT. If the score is > 0.63, this risk score identifies patients with LVEF ≤35% and CRT-D as having an increased likelihood of combined response to CRT.

Equation (1): Probability (*p*) of the combined response to CRT


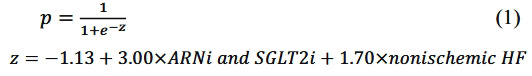


### 3.7 Overall Survival

**Fig. (7):**
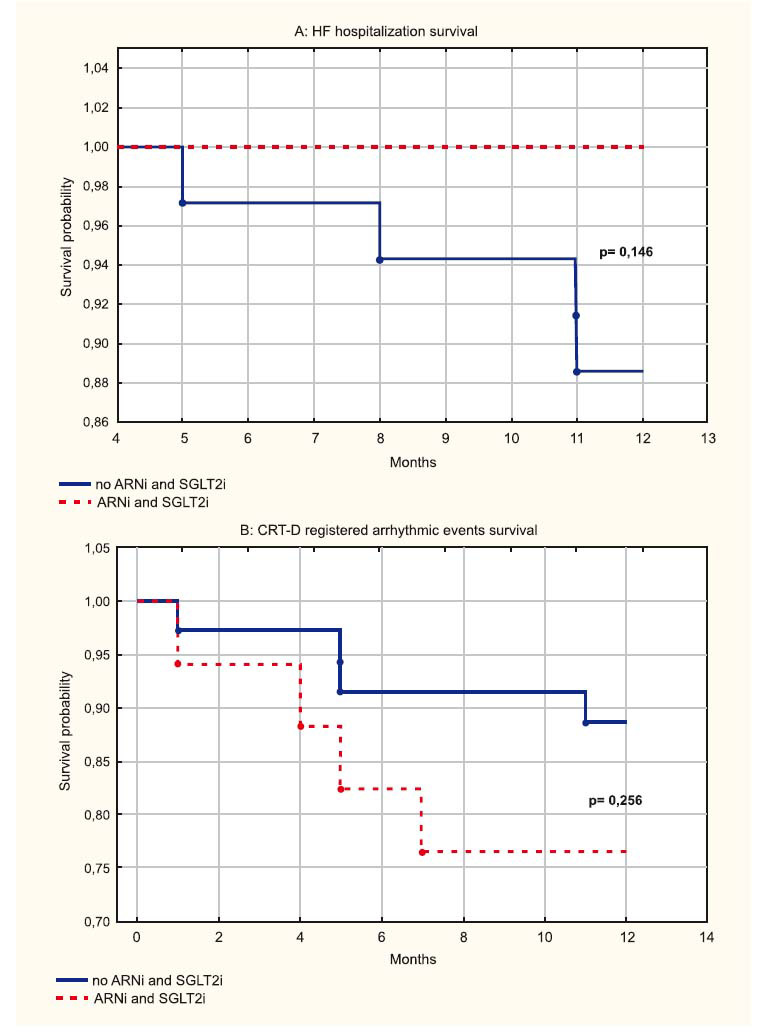
Event-free survival curves according to the groups. Kaplan-Meier curves comparing the risk of (**A**) heart failure (HF) hospitalization and (**B**) CRT-D registered arrhythmic events between group without angiotensin receptor neprilysin inhibitor (ARNi) and sodium-glucose co-transporter 2 inhibitor (SGLT2i) treatment and group with combined ARNi and SGLT2i treatment.

During the 12^th^ months of follow-up, no patients died due to cardiovascular death related to HF. Kaplan-Meier estimates of HF hospitalization and CRT-D registered arrhythmic events in ARNi with SGLT2i combined treatment and no treatment groups are shown in Fig. (**7**). In no ARNi and SGLT2i treatment group during 12^th^ months, 4 (11.4%) patients were hospitalized due to HF, and in ARNi and SGLT2i treatment group, no (0.0%) patient got hospitalized (*p=*0.146) (Fig. **7A**). In no ARNi and SGLT2i group, during 12^th^ months, CRT-D registered arrhythmic events were revealed in 4 (11.4%) patients, and in ARNi and SGLT2i treatment group, they were revealed in 4 (23.5%) patients (*p=*0.256) (Fig. **7B**). All of them had non-sustained VT with spontaneous termination.

## 4 DISCUSSION

### 4.1 Key Findings

In our study, we observed SGLT2i and ARNi combined treatment to be related to a more significant enhancement in cardiac function and a high probability of combined response to CRT in comparison to patients without ARNi and SGLT2i combined treatment in CRT recipients during mid-term follow-up. An increase in ∆LVEF and 6MWDT, and as a result, a significant improvement of NYHA FC, were observed 12^th^ months after ARNi with SGLT2i combination therapy and CRT device implantation. These enhancements were greater than those of CRT recipients who did not receive ARNi and SGLT2i treatment after CRT implantation. These findings propose that the combined treatment with SGLT2i and ARNi may improve clinical outcomes in HF patients with reduced LVEF and CRT implantation indications, and provide additional benefits.

### 4.2 Strengths and Limitations

Our study has some limitations. This research study included relatively small sample and event sizes. Therefore, the results of our work must be interpreted with caution. Also, this was a non-randomized, single-center, retrospective study. Our study did not include data regarding natriuretic peptides, which may have diminished the value of the results.

### 4.3 Comparison with Similar Research Studies and Explanation of Findings

Medical therapy for HF improves symptoms, functional ability, and survival. Nevertheless, despite the optimal medical therapy, many patients with pronounced disturbances in myocardial contractility continue to have symptoms that limit their functionality and quality of life. Many HF patients with reduced LVEF have additional interventricular or intraventricular dyssynchrony, which can further impair cardiac output and which, in some cases, may be the primary cause of LV dysfunction itself [21]. Ventricular dyssynchrony results in adverse changes in ventricular loading and hemodynamics, cardiac blood flow and energy metabolism, gene and protein expression, and valvular regurgitation, eventually culminating in progressive adverse ventricular remodeling, which can cause further deterioration of LV function [22]. CRT targets and treats ventricular dyssynchrony and it has been associated with reduced HF hospitalization, improved functional status, better quality of life, and decreased mortality in multiple randomized trials [23]. At the same time, 30.0-50.0% of patients do not respond to CRT [9]. Many attempts have been made for CRT response optimization, including the implementation of novel therapy. For example, SGLT2i and ARNi have shown significant benefits in patients with HF [24].

Until recently, ACEi/ARB, beta-blockers, and mineralocorticoid receptor antagonists (MRA) have been the mainstay for the management of HF patients with reduced LVEF treatment [25]. However, HF-related hospitalization and mortality remain high, creating a need for the development of new medications [6, 26]. The invention of ARNi and its subsequent use in clinical practice marked a major breakthrough in HF treatment, as it reduced the risk of HF-related hospitalization and death, compared to the treatment with ACEi and ARB [6, 27, 28]. Further studies have reported the prognostic advantages of ARNi to be derived from robust enhancements in TTE indicators [6, 29, 30]. The consecutive cardioprotective impacts of ARNi are associated with neprilysin inhibition [6]. It leads to an increase in vasoactive peptides (natriuretic and glucagon-like peptides, bradykinin, angiotensin I and II). The elevated levels of vasoactive peptides improve glycemic control by increasing insulin sensitivity and metabolism, enhance the mobilization of lipids from adipose tissue, improve muscular oxidative capacity, and enhance adiponectin release [6, 31, 32]. All of these factors are crucial for the pathologic cardiac remodeling. Also, the increased levels of cyclic guanosine monophosphate prevent the loss of protective effects of protein kinase G, which promotes diastolic relaxation, improves ventriculoatrial coupling, and blunts cardiomyocyte stiffness and hypertrophy [6, 31].

Another innovative class of drugs for HF treatment is SGLT2i. The cardiovascular benefits of SGLT2is have been reported in several randomized controlled trials, especially their role in reducing the risk of HF worsening [5, 10-12, 33]. The positive effect of SGLT2is in HF patients is associated with hemodynamic and protective mechanisms. SLGT2i decreases the preload and afterload, and reduces plasma and interstitial volume. In addition, SGLT2i acts on the proximal renal tubule and promotes the reduction in intraglomerular pressure through restored tubule-glomerular feedback [6]. Alleviated renal stress can improve cardiac function through reduced sympathetic nerve system activation, inflammation, and reactive oxygen species generation [6]. Additional protective mechanisms of SGLT2i against HF are thought to be a result of improved efficiency of the myocardial energy metabolism [6, 34, 35].

Since SGLT2i, ARNi, and CRT have different mechanisms of cardioprotective action, a combination of device therapy with these drugs may exhibit synergistic effects. To our knowledge, to date, only one study has evaluated the potential synergy of drugs when administered concurrently with CRT [13, 14]. Currently, in accordance with the latest guidelines for HF diagnosis and treatment, sodium-glucose co-transporter 2 inhibitors and angiotensin receptor neprilysin inhibitors are class I indications in HF patients with reduced LVEF to minimize the risk of HF-related hospitalization and death [7], but a clear indication for combination with CRT is lacking.

Representative trials of sodium-glucose co-transporter 2 inhibitors, such as DAPA-HF and EMPEROR-Reduced trials, have shown consistent benefits in the treatment of HF with reduced LVEF, regardless of the use of ARNi [6, 33, 36, 37]. However, subgroup analysis in these studies has demonstrated synergistic effects of combined ARNi and SGLT2i treatment with CRT. At the same time, Fonderico *et al*. have shown ARNi alone or in combination with SGLT2i in CRT patients to improve the clinical and echocardiographic response at 12 months [13, 14]. To compare the effects of the combination treatment of ARNi and SGLT2i with ACEI/ARB therapy, we divided the patients into two groups based on the use of ARNi with SGLT2i and analyzed the outcomes.

We found patients with non-ischemic HF with reduced LVEF and CRT who received the combination of sodium-glucose co-transporter 2 inhibitors and angiotensin receptor neprilysin inhibitors to have significantly better clinical and echocardiographic outcomes at 12 months of follow-up compared to patients who did not receive either drug. Our results have been found to be consistent with previous studies and further support the idea that ARNi and SGLT2i act through independent mechanisms and provide additional benefits in the treatment of HF [6]. We compared changes in clinical and echocardiographic parameters before and after CRT (6^th^ and 12^th^ months), which indicated response to treatment, contributing to improved prognosis. Improvements in echocardiographic parameters were observed 6^th^ months after CRT implantation. However, patients who received combination therapy of ARNi and SGLT2i had greater improvements compared to those who did not receive either drug. A comparison of 6^th^ months ∆of LVEF between groups did not reveal significant differences (*p=*0.059). However, ∆LVEF at the 12^th^ months in the combined ARNi and SGLT2i treatment group was significantly higher than that in the no-treatment group (*p=*0.045). These findings have been found to be consistent with the results of the study by Fonderico *et al*., in which CRT recipients treated with ARNi and SGLT2i showed significant improvements in left ventricular function compared to patients not receiving either drug in the general population (*p=*0.029) [13, 14]. A comparison of 6^th^ and 12^th^ months 6MWDT in the ARNi and SGLT2i treatment group was significantly better than in the no treatment group (*p<*0.001 and *p=*0.001, respectively). As a result, the number of patients with NYHA class I at 6^th^ and 12^th^ months follow-up in the ARNi and SGLT2i treatment group was significantly higher than in the no treatment group (*p=*0.005 and *p=*0.007, respectively). These data have been found to be partly consistent with the results of the study by Fonderico *et al*. study, in which NYHA FC improved by at least 1 class in 66.7% of the patients in the sodium-glucose co-transporter 2 inhibitors and angiotensin receptor neprilysin inhibitors group *vs.* 42.8% in the no treatment group patients (*p=*0.038) [13, 14].

In our study, based on the definition of a combined response to CRT at 12^th^ month follow-up, 94.1% of patients in sodium-glucose co-transporter 2 inhibitors and angiotensin receptor neprilysin inhibitors treatment group *vs.* 51.4% in the no treatment group were classified as CRT responders (*p=*0.002). Univariable and multivariable logistic regression analyses revealed significant associations between ARNi and SGLT2i treatment and the combined response to CRT (OR=20.09, 95% CI 2.10-192.15, *p=*0.009). These findings have been found to be consistent with the results of other studies. In the studies by Fonderico C. *et al*., CRT response was defined separately with respect to clinical and echocardiographic parameters [13, 14]. In this study, based on the clinical response definition, 88.9% patients in the ARNi and SGLT2i group *vs.* 54.5% in the no treatment group were classified as responders (*p=*0.014). According to the echocardiographic definition, there were more CRT responders in ARNi and SGLT2i group than in the no treatment group (77.7% *vs.* 50%, *p=*0.036). Univariable and multivariable logistic regression tests revealed a significant association between sodium-glucose co-transporter 2 inhibitors and angiotensin receptor neprilysin inhibitors treatment and clinical response (OR=3.72, 95% CI 1.4-10.98, *p=*0.011) [13].

The non-ischemic HF was previously reported to predict reverse remodeling in patients with more advanced HF symptoms. According to Ypenburg *et al*., CRT responders more frequently had non-ischemic HF [38]. In the study by Verhaert *et al*., non-ischemic HF and female gender were associated with a much greater initial response to CRT [39]. According to studies, ischemic HF patients have a diminished capacity for reverse remodeling [39, 40]. This is often attributed to their higher baseline risk with more comorbidity, older age, and more often the presence of myocardial scarring not amenable by CRT [41]. The fibrotic scar tissue in the myocardium after an ischemic event impairs proper conduction of the electrical impulses generated by the CRT device, inhibiting cardiomyocyte contraction and thereby hampering reverse remodeling of the ventricle [42]. In non-ischemic cardiomyopathy, fibrosis may also be present, but its localization is different from ischemic cardiomyopathy, where fibrosis follows subendocardial or transmural distribution along the coronary arteries [42]. In our study, the number of patients with ischemic and non-ischemic HF did not significantly differ in ARNi and SGLT2i treatment and the no treatment groups (*p=*0.882). A comparison of the HF etiology with the grouping of a combined response to CRT had not been made. However, the univariable and multivariable logistic regression analyses have revealed a significant association between the non-ischemic HF and a combined response to CRT (OR=5.51, 95% CI 1.21-24.91, *p=*0.026).

In our study, the combined ARNi and SGLT2i treatment and non-ischemic HF associated with the ventricular remodeling processes were selected for our risk stratification model. These parameters appear to predict the combined response to CRT in these patients, as they are associated with ventricular remodeling processes. The developed risk stratification model allowed us to correctly diagnose the combined response to CRT in our study cohort with an accuracy of 75.0%. The sensitivity of the model was 47.1%, while the specificity was 94.4%. The positive predictive value was 91.2%, while the negative predictive value was 44.4%. Thus, a negative result from our risk stratification model (below the cut-off value) can be used to rule out the combined response to CRT. The negative predictive value is of particular importance in clinical applications. Patients scoring above the cut-off value may benefit more from CRT-D implantation.

ARNi and SGLT2i significantly reduced the risk of HF-related hospitalization or death in HF patients with reduced LVEF [28, 29, 43]. Results from two independent studies (DAPA-HF and EMPEROR-Reduced) showed the effect of SGLT2i on HF hospitalizations to be consistent and suggested that these drugs also reduce all-cause and cardiovascular mortality in HF patients with reduced LVEF [5, 44]. Among 8474 patients enrolled in the DAPA-HF and EMPEROR-Reduced study, the estimated effect of SGLT2i treatment was a significant 26% relative reduction in all-cause cardiovascular mortality and first HF hospitalization [37]. In a post-hoc analysis of DAPA-HF, during 18 months follow-up, SGLT2i reduced the risk of the primary endpoint of any life-threatening ventricular tachyarrhythmias, cardiac arrest, or sudden cardiac death, and the effectiveness appeared to be more substantial in patients without an ICD device, although the interaction with SGLT2i was not significant [45]. According to the authors, the device cohort was of modest size with relatively few events, but above all, patients with CRT and ICD were considered together, which did not allow evaluating the possible synergic effect of CRT and SGLT2i on myocardium remodeling and ventricular tachyarrhythmias. Conversely, a study by Sfairopoulos *et al*. failed to show a significant relation between SGLT2i and the risk of sudden cardiac death or ventricular tachyarrhythmias [46]. The work carried out by Kim *et al*. showed that patients receiving ARNi and SGLT2i therapy, compared to individuals without therapy, had a significantly lower risk of cardiovascular death [hazard ratio (HR): 0.18; 95% CI: 0.53-0.61; *p=*0.00^6^] and HF-related hospitalization (HR: 0.42; 95% CI: 0.22-0.82; *p=*0.011) [6]. In our event-free survival analysis, Kaplan-Meier curves did not demonstrate an analogous tendency in estimating survival free of HF hospitalization, CRT-D registered arrhythmic events, and appropriate ICD-therapy events according to combined ARNi with SGLT2i treatment.

The role of ARNi and SGLT2i separately or in combination in acute HF patients with different hemodynamic phenotypes of HF has been poorly studied. Today, acute decompensated HF is viewed as a complex heterogeneous clinical syndrome, with classifications that rely heavily on non-specific descriptors, such as LVEF cut-off points (HF with preserved *vs.* reduced LVEF) and hemodynamic profiles based on bedside assessments of cardiac output (“cold” *vs.* “warm”) and filling pressures (“wet” *vs.* “dry”) [47, 48]. Currently, there are four hemodynamic phenotypes of HF: “warm” (no signs or symptoms of hypoperfusion) and “dry” (no signs or symptoms of congestion), “warm” (no hypoperfusion) and “wet” (presence of any signs or symptoms of congestion), “cold” (presence of any signs or symptoms of hypoperfusion) and “wet” (congestion), and “cold” (hypoperfusion) and “dry” (no congestion). These HF profiles have been adapted to advance HF from Forrester-Diamond classifications of congestion and perfusion estimates in acute myocardial infarction [48, 49]. According to Zhou *et al*., the use of sacubitril-valsartan sodium in patients with acute myocardial infarction complicated with HF can significantly improve cardiac function and reverse ventricular remodeling, reducing the risk of HF re-hospitalization [50]. Carvalho *et al*. showed that the addition of SGLT2i to conventional therapy for acute HF reduced all-cause death (OR: 0.75; 95% CI: 0.56-0.99; *p* = 0.049), readmissions for HF (OR: 0.54; 95% CI: 0.44-0.66; *p* < 0.001), and the composite of cardiovascular death and HF readmissions (hazard ratio: 0.71; 95% CI: 0.60-0.84; *p* < 0.001) [51]. Furthermore, SGLT2i increased mean daily urinary output in liters [mean difference (MD): 0.45; 95% CI: 0.03-0.87; *p* = 0.035) and decreased mean daily doses of loop diuretics in mg of furosemide equivalent (MD: -34.90; 95% CI: - 52.58, - 17.21; *p* < 0.001) without increasing the incidence of worsening renal function (OR: 0.75; 95% CI: 0.43-1.29; *p* = 0.290) [51]. The pooled analysis of 1,347 patients (881 from PIONEER-HF, 466 from PARAGLIDE-HF) demonstrated that in patients stabilized after recent worsening heart failure, sacubitril/valsartan led to a greater reduction in plasma NT-proBNP (n = 1,130; ratio of change = 0.76; 95% CI: 0.69-0.83; *p* < 0.0001) and improved clinical outcome (HR: 0.70; 95% CI: 0.54-0.91; *P* = 0.0077) compared to control therapy, in particular across the spectrum of LVEF ≤60% [52]. In the study by Chen *et al*., patients with sacubitril/valsartan treatment had a lower risk of HF re-hospitalization (HR: 0.83, 95% CI: 0.74-0.92) and all-cause death (HR: 0.51, 95% CI: 0.27-0.94) compared to ACEI/ARB therapy [53]. The impact of ARNi and SGLT2i combined treatment on hemodynamic phenotypes of HF is not described in the available literature.

HF hemodynamic categories are poorly characterized in the elderly. In the retrospective study performed by Sonaglioni *et al*., male sex, “cold-dry” phenotype, high sodium level, and low glomerular filtration rate (GFR) were reported as the main adverse prognostic indicators over a mid-term follow-up in hospitalized patients aged ≥ 70 years [54]. An extremely important question is the possibility to use ARNi and SGLT2i in the “cold” and “dry” phenotype that is commonly detected in elderly patients hospitalized with HF. Since our study included patients with an average age of 60 years, the issue of using these drugs is relevant. According to studies involving elderly HF patients with reduced LVEF, treatment with ARNi and SGLT2i seems effective and safe. Thus, the improvements in LVEF and cardiac remodeling, blood pressure (BP), GFR, serum glucose, uric acid, and glycated hemoglobin could be the mechanisms by which ARNi and SGLT2i play their beneficial role in clinical outcomes. In confirmation of this, Mazza *et al*. showed that in elderly patients with reduced LVEF, treatment with ARNi improved LVEF and GFR levels compared to controls (42.4 *vs.* 34.2%, 73.8 *vs.* 61.2 mL/min, respectively; *p* < 0.001) [55]. NT-proBNP, clinic systolic and diastolic BP, blood glucose, glycated hemoglobin, and uric acid values were reduced in both treatment arms, but they were lower in the ARNi group compared to controls (3107 *vs.* 4552 pg/mL, 112.2 *vs.* 120.4, and 68.8 *vs.* 75.6 mmHg; 108.4 *vs.* 112.6 mg/dL, 5.4 *vs.* 5.9%, and 5.9 *vs.* 6.4 mg/dL, respectively; *p* < 0.05) [55]. Mortality and HF rehospitalization were lower in the ARNi group than in controls (20.1 *vs.* 33.6% and 27.7 *vs.* 46.3%, respectively; *p* < 0.05) [55]. The OASIS-HF study demonstrated SGLT2i treatment to be associated with the reduction in the composite events of HF rehospitalization or cardiovascular death and protect against worsening renal function, along with leading to a decrease in long-term repeated HF rehospitalizations in patients aged ≥75 years hospitalized for acute decompensated HF [56]. In our study, event-free survival analysis did not demonstrate an analogous tendency in estimating survival free of HF hospitalization according to combined ARNi with SGLT2i treatment due to a relatively small sample size and short follow-up time.

### 4.4 Implications and Actions Required

In CRT patients, combination therapy with ARNi and SGLT2i is recommended to achieve better clinical outcomes. Further large sample-size research studies are needed to verify if this treatment can lead to improved outcomes and survival over long-term follow-up.

## CONCLUSION

The combined treatment with SGLT2i and ARNi in CRT recipients enhanced the clinical and echocardiographic response at the 12^th^-month follow-up. In our cohort of patients, the response to CRT was associated with non-ischemic HF and combined ARNi and SGLT2i therapy. Our developed risk model including these two variables could discriminate individuals with a combined CRT response. The combined ARNi and SGLT2i treatment had no significant effect on cardiovascular HF-related mortality, HF hospitalization, and CRT-D-registered arrhythmic events.

## Figures and Tables

**Table 1:** Patients’ demographic and clinical characteristics in the overall population and by group.

**Demographic and Clinical Characteristics**	**Overall Population (n=52)**	**1^st^ Group (no ARNi and SGLT2i) (n=35)**	**2^nd^ Group (ARNi and SGLT2i) (n=17)**	**p_2-3_**
	1	2	3	
Age, year, M ± SD	60.0 ± 10.0	60.4 ± 11.0	59.1 ± 7.8	0.618
Male gender, n (%)	34 (65.4)	22 (62.8)	12 (70.6)	0.582
***Heart failure etiology***				
Ischemic, n (%)	16 (30.7)	11 (31.4)	5 (29.4)	0.882
Non-ischemic, n (%)	36 (69.3)	24 (68.6)	12 (70.6)	0.882
***Baseline New York Heart Association class***				
II, n (%)	20 (38.4)	12 (34.3)	8 (47.0)	0.374
III, n (%)	32 (61.6)	23 (65.7)	9 (53.0)	0.374
Baseline 6-minute walk distance test, m, M_e_ (Q_1_; Q_3_)	295.0 (250.0; 325.0)	290.0 (250.0; 310.0)	320.0 (275.0; 350.0)	0.418
***Arrhythmias registered prior to CRT-D implantation***				
History of sustained VT, n (%)	2 (3.8)	1 (2.8)	1 (5.9)	0.594
Paroxysmal AF, n (%)	25 (48.1)	18 (51.4)	7 (41.2)	0.487
***Comorbidities***				
Hypertension, n (%)	16 (30.7)	11 (31.4)	5 (29.4)	0.882
Diabetes mellitus, n (%)	12 (23.1)	7 (20.0)	5 (29.4)	0.449
Baseline BMI, kg/m^2^, M ± SD	28.8 ± 4.6	28.3 ± 4.2	29.7 ± 5.4	0.334
Dyslipidemia, n (%)	31 (59.6)	21 (60.0)	10 (58.8)	0.935
Initial eGFR, ml/min/1.73 m^2^, M ± SD	71.6 ± 18.3	70.9 ± 17.6	73.2 ± 20.0	0.718
Stroke, n (%)	2 (3.8)	1 (2.8)	1 (5.9)	0.594
***Initial electrocardiographic findings***				
LBBBs, n (%)	45 (86.5)	30 (85.7)	15 (88.2)	0.802
Right bundle branch block, n (%)	7 (13.5)	5 (14.3)	2 (11.8)	0.802
PQ interval, ms, M ± SD	194.7 ± 41.0	188.5 ± 41.4	208.5 ± 37.7	0.060
QRS duration, ms, M_e_ (Q_1_; Q_3_)	171.0 (156.0; 182.0)	164.0 (154.0; 180.0)	174.0 (162.0; 188.0)	0.103
QT_c_ (LBBB/BP), ms, M ± SD	423.9 ± 35.8	415.8 ± 37.8	440.9 ± 24.6	0.019
Alpha angle, °, M_e_ (Q_1_; Q_3_)	-30.0 (-46.0; 20.0)	-30.0 (-45.0; 30.0)	-30.0 (-47.0; -5.0)	0.545
BP at 12^th^ month, %, M ± SD	97.9 ± 1.7	97.9 ± 1.6	97.7 ± 2.1	0.992
***Baseline echocardiographic findings***				
Left ventricular end-systolic volume, ml, M_e_ (Q_1_; Q_3_)	180.0 (127.0; 209.5)	175.0 (128.0; 208.0)	186.0 (126.0; 210.0)	0.612
Left ventricular ejection fraction, %, M_e_ (Q_1_; Q_3_)	28.0 (22.0; 31.0)	28.0 (22.0; 31.0)	28.0 (25.0; 31.0)	0.605
***Therapy***				
Beta-blockers, n (%)	51 (98.1)	35 (100.0)	16 (94.1)	0.147
Loop diuretics, n (%)	43 (82.7)	28 (80.0)	15 (88.2)	0.461
MRA, n (%)	46 (88.4)	30 (85.7)	16 (94.1)	0.373
ACEI, n (%)	21 (40.4)	21 (60.0)	0 (0.0)	<0.001
Antiplatelet agents, n (%)	19 (36.5)	13 (37.1)	6 (35.3)	0.896
Lipid-lowering treatment, n (%)	41 (78.8)	26 (74.3)	15 (88.2)	0.247
Angiotensin II receptor blocker, n (%)	14 (26.9)	14 (40.0)	0 (0.0)	0.002
Amiodarone, n (%)	26 (50.0)	19 (54.3)	7 (41.2)	0.375
Anticoagulants, n (%)	34 (41.4)	19 (54.3)	9 (53.0)	0.927
Ivabradine, n (%)	1 (1.9)	0 (0.0)	1 (5.9)	0.147
***Left ventricular lead position***				
Lateral vein, n (%)	23 (44.2)	14 (40.0)	9 (53.0)	0.378
Posterolateral vein, n (%)	18 (34.6)	13 (37.1)	5 (29.4)	0.582
Anterolateral vein, n (%)	11 (21.2)	8 (22.9)	3 (17.6)	0.666

**Note:** Data are expressed as n (%) for categorical and qualitative variables, and M ± SD or M_e_ (Q_1_; Q_3_) for continuous variables. **Abbreviations:** ACEI: angiotensin-converting enzyme inhibitors, AF: atrial fibrillation, ARNi: angiotensin receptor neprilysin inhibitor, BMI: body mass index, BP: biventricular pacing, CRT-D: cardiac resynchronization therapy device with the defibrillator, eGFR: estimated glomerular filtration rate, LBBBs: left bundle branch block meeting Strauss criteria, MRA: mineralocorticoid receptor antagonist, QT_c_ (LBBB/BP): corrected QT_c_ interval adjusted for LBBB/BP, SGLT2i: sodium-glucose co-transporter 2 inhibitor, VT: ventricular tachycardia.

## Data Availability

The authors confirm that the data supporting the findings of this research are available within the article.

## LIST OF ABBREVIATIONS

ACEi: Angiotensin-Converting Enzyme Inhibitors
AF: Atrial Fibrillation
ARB: Angiotensin II Receptor Blockers
ARNi: Angiotensin Receptor Neprilysin Inhibitors
ATP: Anti-Tachycardia Pacing
AUC: Area Under the Curve
BP: Biventricular Pacing
CI: Confidence Intervals
CRT: Resynchronization Therapy
HF: Heart Failure
LVESV: Left Ventricular End-Systolic Volume
MRA: Mineralocorticoid Receptor Antagonists
NYHA: New York Heart Association
TTE: Transthoracic Echocardiography

## STANDARDS OF REPORTING

STROBE guidelines were followed.
